# The complete plastid genome sequence of *Lysidice brevicalyx* (Fabaceae: Detarioideae), an arbor species endemic to China

**DOI:** 10.1080/23802359.2023.2259041

**Published:** 2023-09-21

**Authors:** Jian-Xin Li, Ying Meng, Ze-Long Nie, Tie-Yao Tu

**Affiliations:** a College of Biology and Environmental Sciences, Jishou University, Jishou, Hunan, China; b Plant Science Center, South China Botanical Garden, Chinese Academy of Sciences, Guangzhou, China; c South China National Botanical Garden, Guangzhou, China

**Keywords:** Phylogenetic analysis, plastid genome, Fabales, *Lysidice*, Leguminosae

## Abstract

The plastid genome of *Lysidice brevicalyx* was successfully assembled using Illumina sequencing reads for the first time. The complete plastid genome of *L. brevicalyx* is a circular structure of 159,084 bp with a GC content of 36.4%. It comprises a large single-copy (LSC) region of 87,783 bp, a small single-copy (SSC) region of 19,557 bp, and two inverted repeat regions (IRA and IRB) of 25,872 bp, each. The plastome of *L. brevicalyx* contains a total of 128 genes, including 83 protein-coding genes, 37 tRNAs, and 8 rRNAs. The phylogenetic analysis strongly supports the monophyly of *Lysidice*. This study provides the first complete plastid genome sequence of *L. brevicalyx* and contributes to our understanding of the molecular characteristics and evolutionary relationships of this plant species.

## Introduction

**Figure 1.**
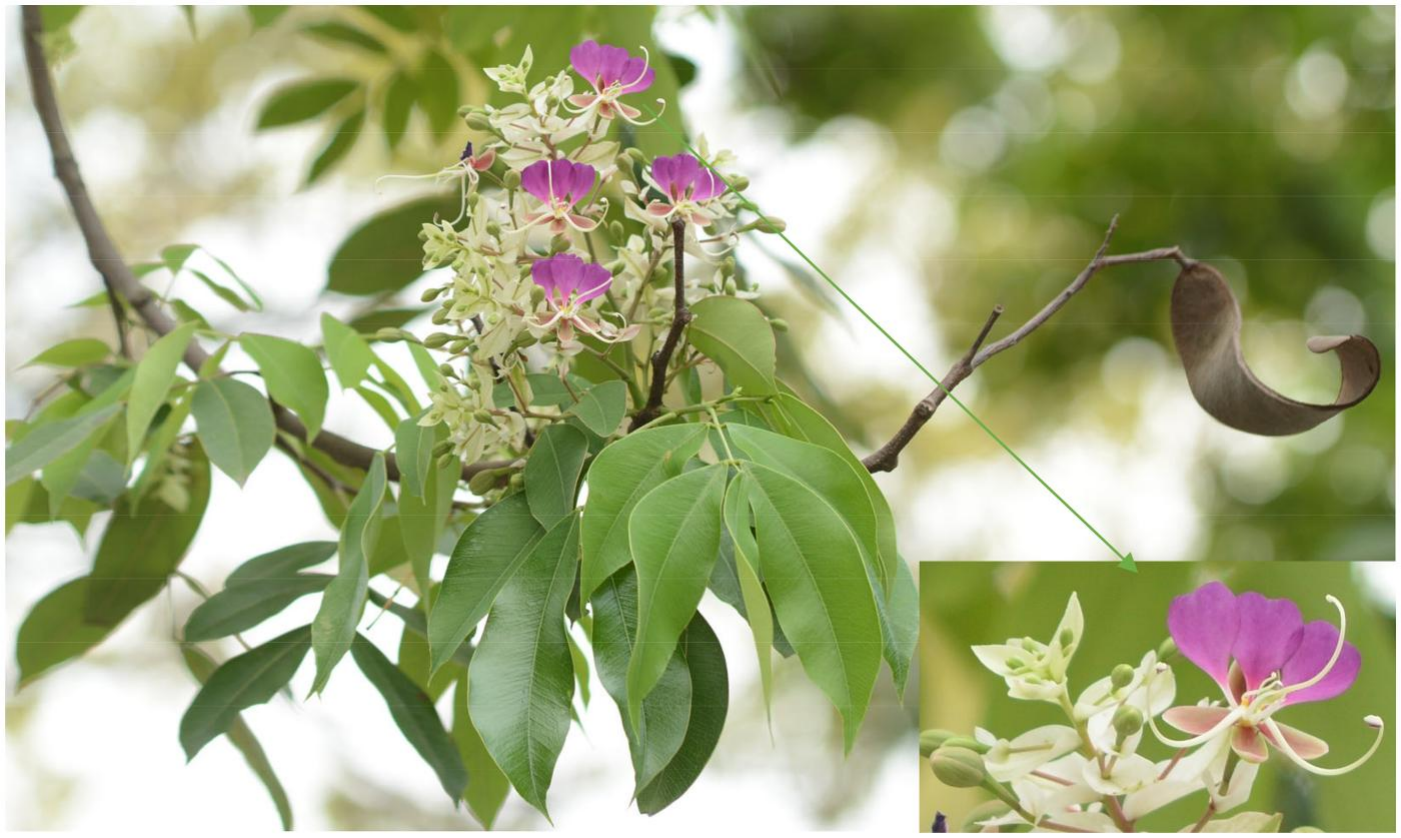
Plant image of *Lysidice brevicalyx*. The image showing flowers are actinomorphic, with purple flag and wing petals, an evenly pinnate leaf, flattened, twisted pods. This photo was taken by youpai zeng.

**Figure 2.**
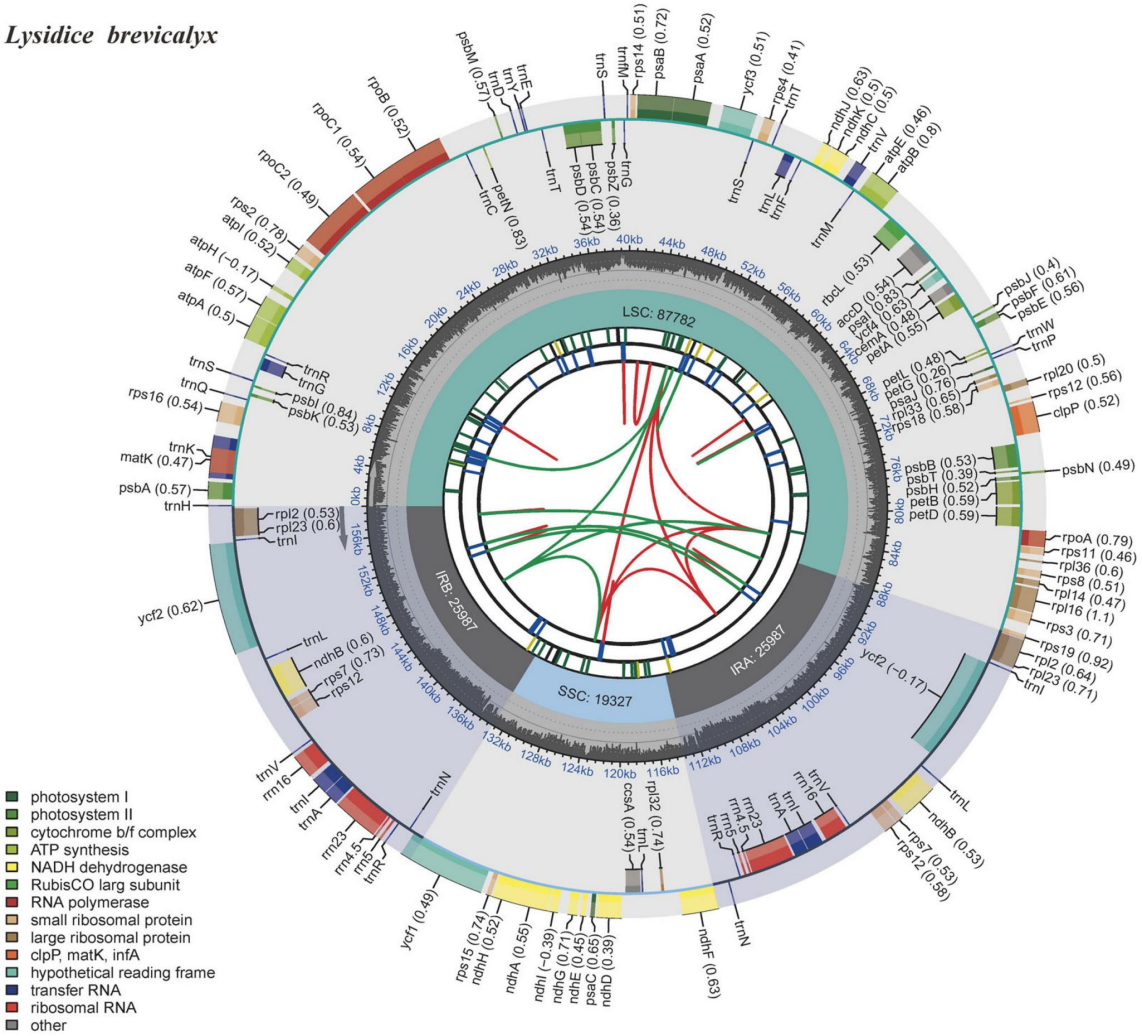
Gene map of the plastid genome of *Lysidice brevicalyx*. From the center outward, the first track indicates the dispersed repeats; the second track shows the long tandem repeats as short blue bars; the third track shows the short tandem repeats or microsatellite sequences as short bars with different colors; the fourth track shows small single-copy (SSC), inverted repeat (IRA and IRB), and large single-copy (LSC) regions. The GC content along the genome is plotted on the fifth track; the genes are shown on the sixth track.

The genus *Lysidice* (Fabaceae) consists of two species, *viz. Lysidice rhodostegia* Hance 1867 from China and Vietnam and *Lysidice brevicalyx* C. F. Wei 1983 endemic to southern China (Hou 2010). *L. brevicalyx* is a valuable tree species used for building materials due to its white-yellow wood (Figure 1) and has been used as an alternative medicine for the treatment of fractures and traumatic bleeding in China (Gao et al. 2007). Additionally, the roots, stems, and leaves of *L. brevicalyx* have been used for medicinal purposes by local communities (Wu et al. 2010, Yang et al. 2019). In this study, we report the first assembly and annotation of the complete plastid genome of *L. brevicalyx* (Figure 2) using second-generation sequencing technology.

## Materials and methods

Fresh leaves of *L. brevicalyx* were collected from the South China Botanical Garden in Guangzhou City, Guangdong Province, China (N 23°11'12.62′', E 113°21'51.15′'). A voucher specimen (*Q. Lai LaiQ036*) was deposited in IBSC (contact person: Qiang Lai, laiqiang@scbg.ac.cn). Total genomic DNA was extracted from the fresh leaves using a combination of the improved cetyltrimethylammonium bromide (CTAB) method (Doyle and Doyle 1987, Yang et al. 2014) and the Dneasy Plant Mini Kit extraction. Quality monitoring was performed using a Qubit 3.0 fluorometer. Genomic DNA was sheared to prepare a PCR-free library with an insert size of 150 bp. High-throughput sequencing was conducted using the Illumina Hiseq X-Ten system, generating a total of 2.3 GB of pair-end reads. The plastid genome was assembled using the GetOrganelle v1.7.7.0 (Jin et al. 2020), and the unique genes of the *L. brevicalyx* plastid genome were annotated using CPGAVAS2 web service (Shi et al. 2019). A gene graphical map of the plastid genome was constructed using CPGVIEW (http://www.1kmpg.cn/ cpgview) (Liu et al. 2023). Then final plastid genome of *L. brevicalyx* was submitted to NCBI Gene Bank with an accession number of OQ808806. Firstly, a total of 19 plastid genomes were downloaded from NCBI Gene Bank, 74 protein-coding genes shared by all genomes were screened. Subsequently, MAFFT v7.313 (Katoh et al. 2019) was used for separate alignment of each gene, and maximum likelihood phylogenies were inferred using IQ-TREE (Minh et al. 2022) under the GTR + F + I + G4 model for 5000 ultrafast bootstraps.

## Results and discussion

The newly assembled plastid genome of *Lysidice brevicalyx* is 159,084 bp in length. The sequencing coverage depths of the genome ranged from **7× to 1873×** with a mean coverage depth of **1195.94×**, indicating the reliability of the genome assembly. The plastid gene structure of *L. brevicalyx* is a circular molecule (Figure 2), consisting of four parts: a large single-copy region (LSC) of 87,783 bp, a small single-copy region (SSC) of 19,557 bp, and two inverted repeat regions (IRA and IRB), each 25,872 bp. The plastome contains a total of 128 genes, including 83 protein-coding genes, 37 tRNAs, 8 rRNAs. Among the protein-coding genes, 14 protein-coding genes (*atp*F, *ndh*A, *ndh*B, *pet*B, *pet*D, *rpl*16, *rpo*C1, *rps*16, *trn*A-UGC, *trn*G-UCC, *trn*I-GAU, *trn*K-UUU, *trn*L-UAA, and *trn*V-UAC) contain one intron and three genes (*clp*P, *rps*12, and *ycf*3) have two introns. Three small-exon genes (*pet*B, *pet*D, and *rps*16) and one trans-spliced gene (*rps*12) were verified to be corrected and annotated with multiple sequence alignment.

**Figure 3.**
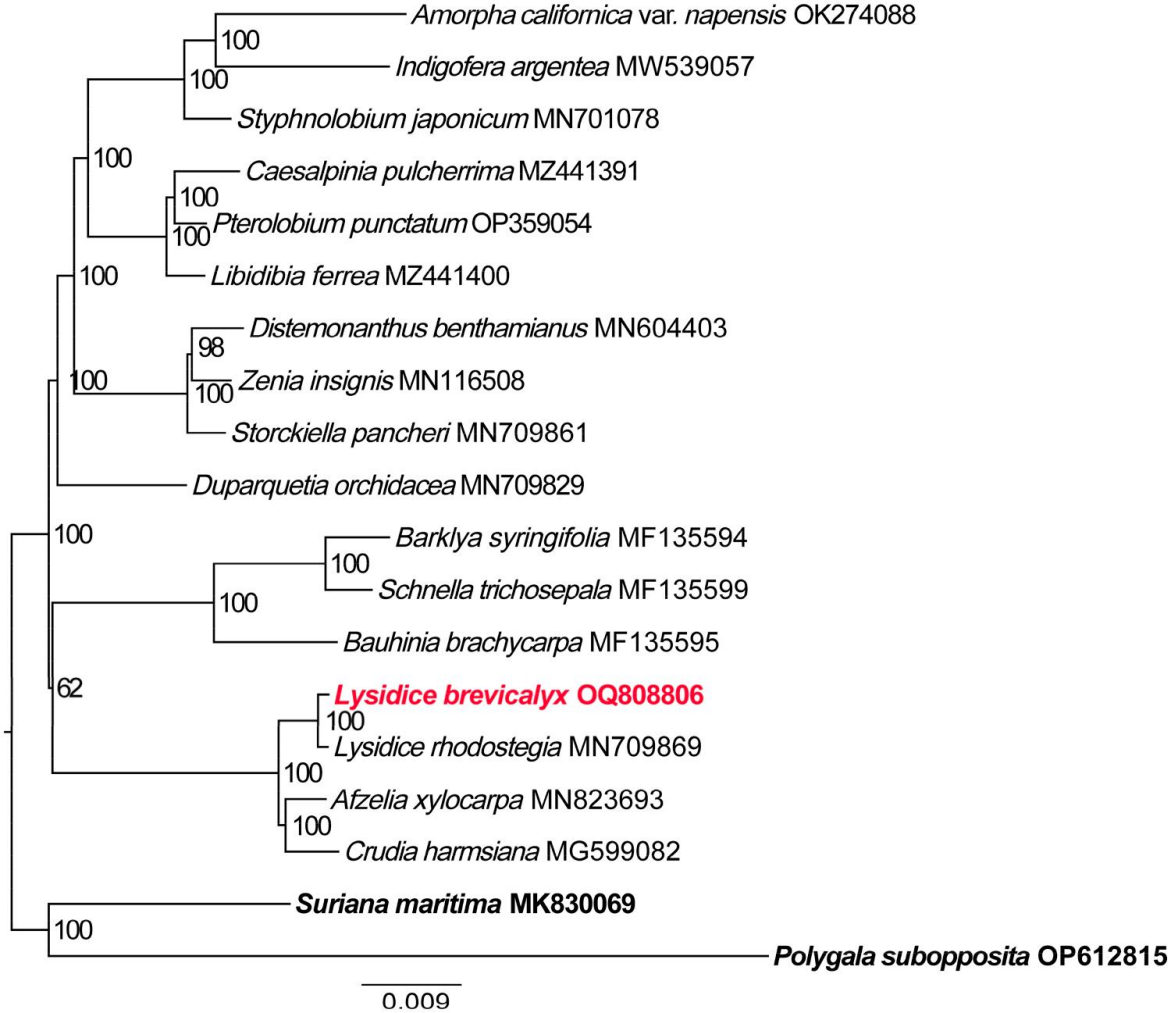
The phylogenetic position for *Lysidice brevicalyx* according to the ML phylogenetic tree constructed based on74 plastid genomes. The following sequences were used: *Polygala subopposita* OP612815 and *suriana maritima* MK830069 (Lai et al. 2019b) as outgroup, *Crudia harmsiana* MG599082 (Tosso et al. 2018), *Afzelia xylocarpa* MN823693 (Zhang et al. 2020a), *Lysidice rhodostegia* MN709869 (Zhang et al. 2020b), *bauhinia brachycarpa* MF135595 (Wang et al. 2018), *schnella trichosepala* MF135599 (Wang et al. 2018) *Barklya syringifolia* MF135594 (Wang et al. 2018), *duparquetia orchidacea* MN709829 (Zhang et al. 2020b), *storckiella pancheri* MN709861 (Zhang et al. 2020b), *zenia insignis* MN116508 (Lai et al. 2019a) *distemonanthus benthamianus* MN604403 (Demenou et al. 2020), *libidibia ferrea* MZ441400 (Aecyo et al. 2021), *pterolobium punctatum* OP359054 (Zhang et al. 2020b), *caesalpinia pulcherrima* MZ441391 (Aecyo et al. 2021) *styphnolobium japonicum* MN701078 (Shi and Liu 2019), *indigofera argentea* MW539057 *amorpha californica* var*. napensis* OK274088 (Agudelo et al. 2022) The sequences used for the tree structure are coding sequences, and the bootstrap support values are shown on the nodes. The scale bar represents the numbers of substitutions per site.

For the construction of the Maximum Likelihood (ML) phylogenetic tree, a total of 19 complete plastid genomes of fabales were used (Figure 3). The result showed that *L. brevicalyx* is closely related to *L. rhodostegia*, *Afzelia xylocarpa* (Kurz) Craib, and *Crudia harmsiana* De Wil, and all of them formed a clade with high supported value (BP = 100%). These findings are consistent with previous research (Zhao et al. 2021). The clade comprising subfamilies Cercidoideae and Detarioideae is sister to the remaining legumes, and Duparquetioideae and Dialioideae are successive sisters to the clade of Papilionoideae and Caesalpinioideae.

## Conclusion

This study presents the first report of the plastid genome of *Lysidice brevicalyx*, and adds to the limited number of plastid genomes reported for the genus *Lysidice*. The phylogenetic analysis revealed that *Lysidice* forms an independent sister clade to other species, providing novel insights into the phylogenetic relationships within the Leguminosae family. The genetic resource information generated in this study will be valuable for further investigations into the biology and evolutionary history of *Lysidice*.

## Supplementary Material

Supplemental Material

## Data Availability

The complete plastid genome sequence of *L. brevicalyx* has been deposited in the NCBI GenBank database under the accession number OQ808806 (these numbers were automatically generated by NCBI and refer to the same sample). The associated BioProject, SRA, and Bio-Sample numbers are PRJNA955444, SRR24167046, and SAMN34178680 respectively.
