# Supplementary material for: The complete plastid genome sequence of Lysidice brevicalyx (Fabaceae: Detarioideae), an arbor species endemic to China
Source: Mitochondrial DNA B Resour. 2023 Sep 21;8(9):1003–6. doi: 10.1080/23802359.2023.2259041 (PMC10515688; doi:10.1080/23802359.2023.2259041)
Supplement: Supplemental Material [file TMDN_A_2259041_SM4983.docx]

The complete plastid genome sequence of *Lysidice brevicalyx* (Fabaceae: Detarioideae), an arbor species endemic to China

Jian-Xin Li^1,2,3^, Ying Meng^1*^, Ze-Long Nie^1^ & Tie-Yao Tu^2,3^

^1^College of Biology and Environmental Sciences, Jishou University, Jishou, Hunan, 416000, China

^2^Plant Science Center, South China Botanical Garden, Chinese Academy of Sciences, Guangzhou 510650, China

^3^South China National Botanical Garden, Guangzhou 510650, China


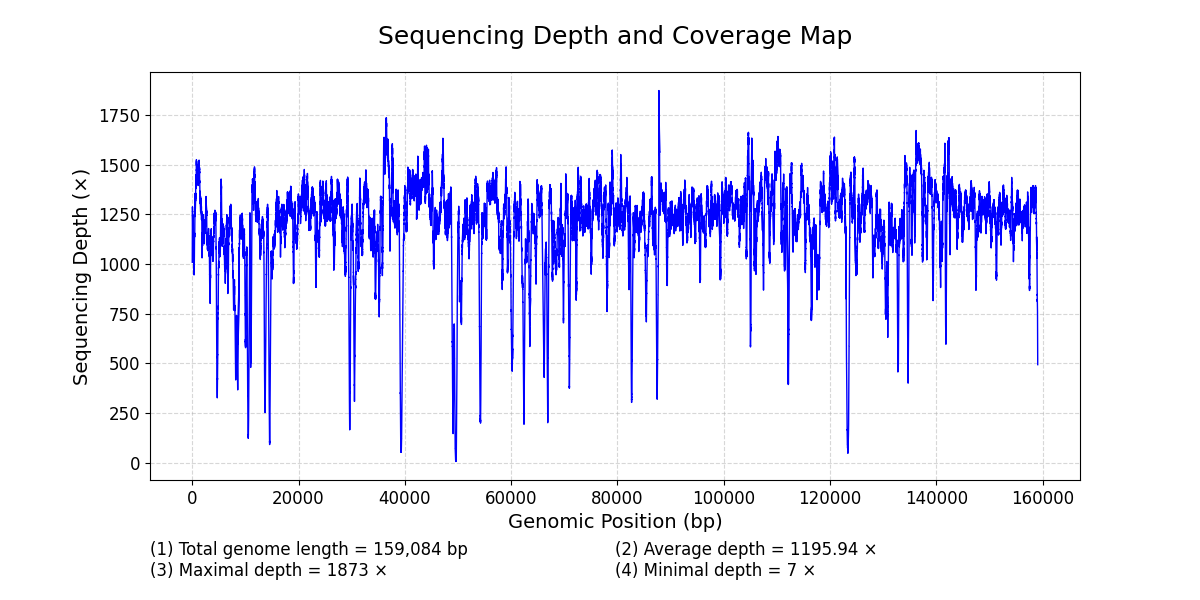


**AA**

**
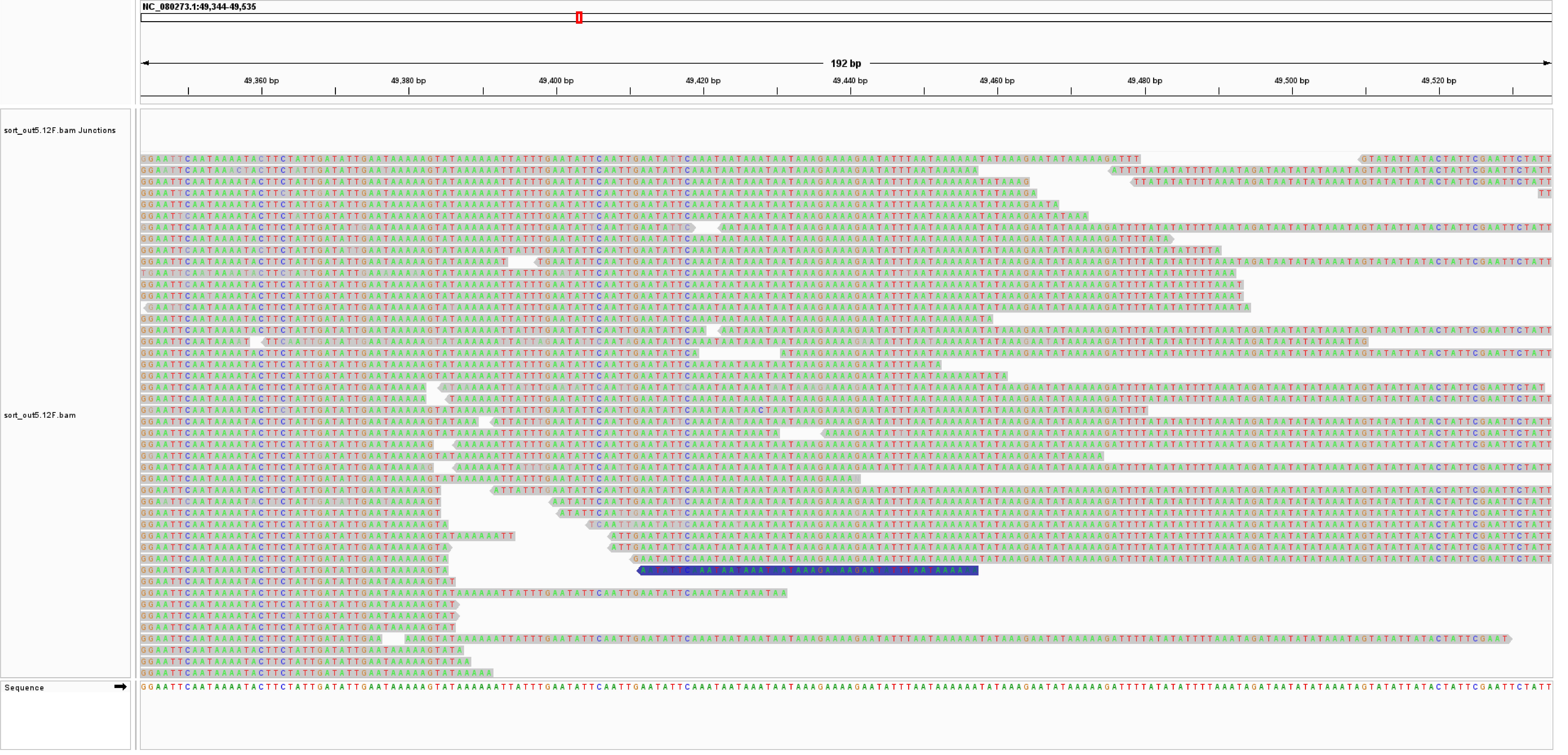

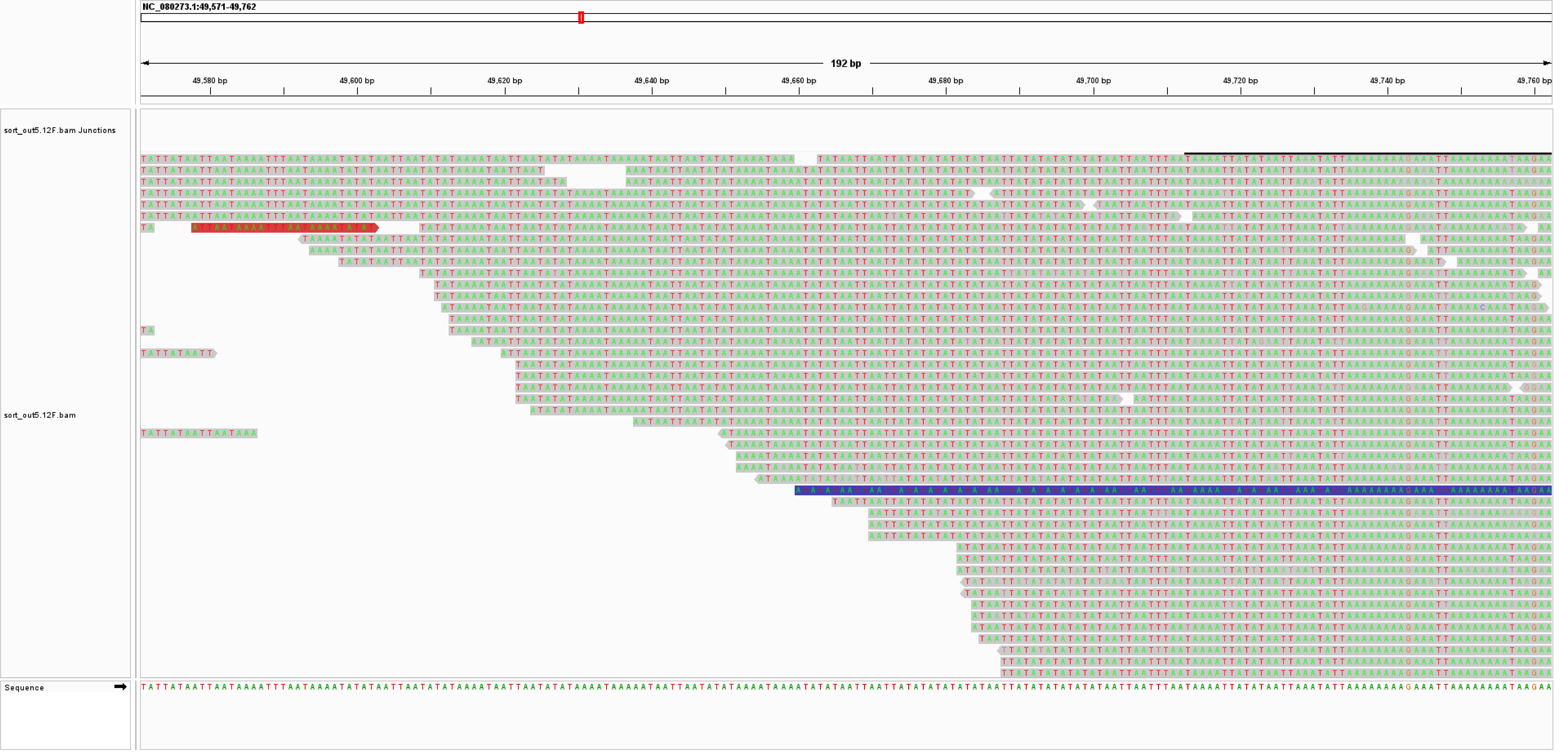
**

**CB**

**BB**

**Supplementary Figure 1.** **(A)** Coverage depth figure of the *Lysidice brevicalyx* chloroplast genome. The horizontal coordinate is the base of the chloroplast genome and the vertical coordinate is the depth of sequencing corresponding to that base.

**(B) (C)** The mapping results between the specific reads and the lower-depth local of the *Lysidice brevicalyx* chloroplast genome


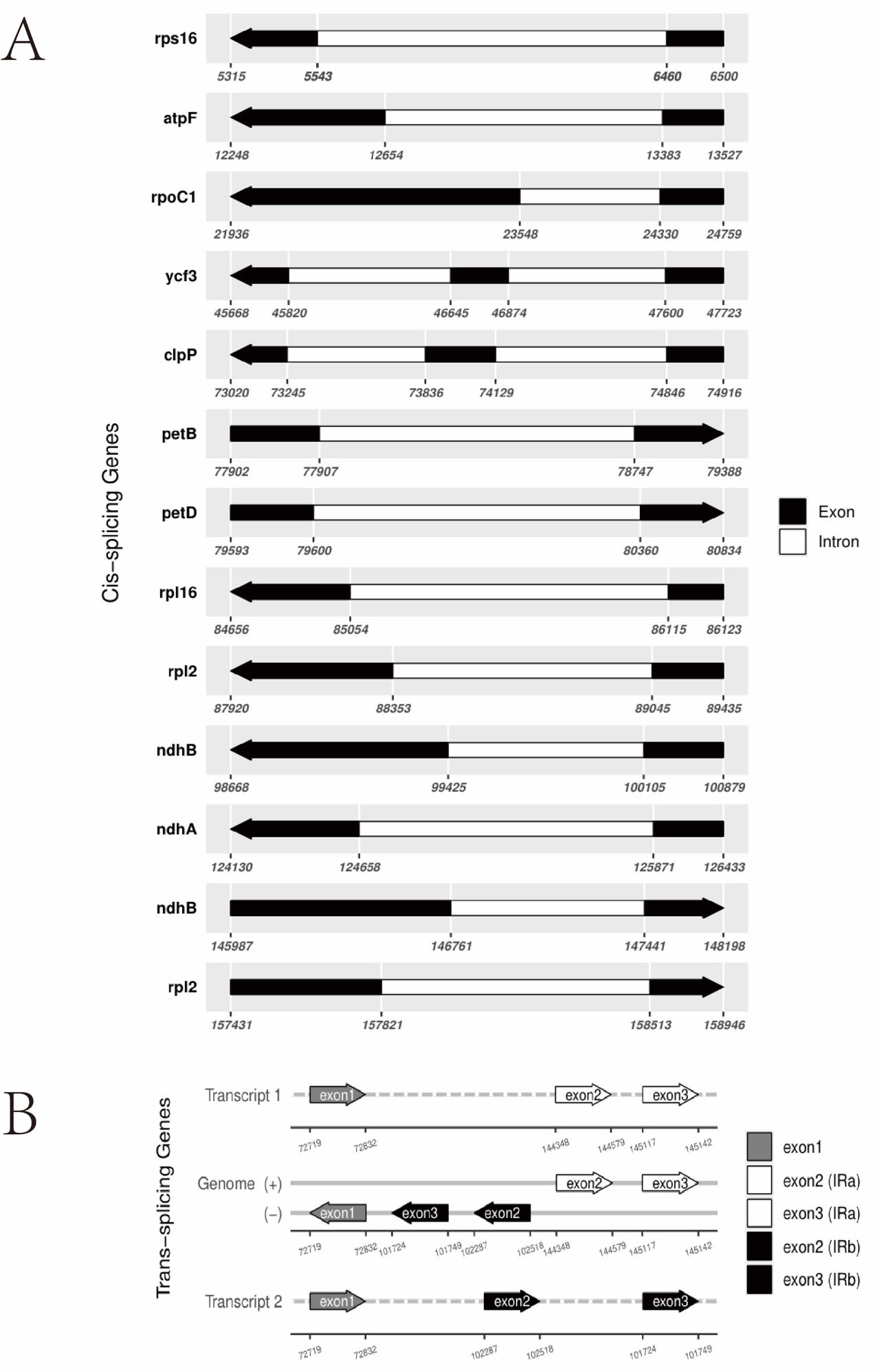


**Supplementary Figure 2.** A. Schematic map of the cis-splicing genes in the *Lysidice brevicalyx* chloroplast genome. B. Schematic map of the trans-splicing gene rps12 in the chloroplast genome.
